# Trends in Patient-Reported Physical Function After Hip Fracture Surgery

**DOI:** 10.7759/cureus.64572

**Published:** 2024-07-15

**Authors:** Parimal Rana, Jane C Brennan, Andrea H Johnson, Paul J King, Justin J Turcotte

**Affiliations:** 1 Orthopedic Research, Anne Arundel Medical Center, Annapolis, USA; 2 Orthopedics, Anne Arundel Medical Center, Annapolis, USA; 3 Orthopedic Surgery, Anne Arundel Medical Center, Annapolis, USA; 4 Orthopedic and Surgical Research, Anne Arundel Medical Center, Annapolis, USA

**Keywords:** postoperative mortality, postoperative function, promis scores, physical function, geriatric hip fracture

## Abstract

Background

Hip fractures carry significant morbidity and mortality, yet studies assessing post-surgical functional recovery from the patient’s perspective are scarce, lacking benchmarks against age-matched populations. This study aimed to identify factors influencing postoperative functional outcomes, compared to the lower 25th percentile of normal age-matched populations, and to compare postoperative physical function with one-year mortality following hip fracture surgery.

Methodology

A retrospective review of 214 hip fracture patients reporting to the emergency department (ED) from July 2020 to June 2023 was conducted, with all completing a three-month postoperative Patient-Reported Outcomes Measurement Information System-Physical Function (PROMIS-PF) survey. Primary outcomes included three-month PROMIS-PF scores, with secondary outcomes focusing on one-year mortality. Factors such as demographics, comorbidities, procedures, time to surgery, length of stay, and postoperative outcomes were analyzed for correlation. Multivariate logistic regression assessed predictors of achieving a PROMIS-PF T-score of at least 32.5, representing the bottom 25th percentile for age-matched populations, and the relationship between three-month PROMIS PF T-scores and one-year mortality.

Results

Surgery was performed within 24 hours of ED arrival in 118 (55.1%) patients, the average length of stay was 5.2 days, and 64 (29.9%) were discharged home. Total hip arthroplasty and home discharge correlated with higher physical function scores. In contrast, older age, higher American Society of Anesthesiologists scores, certain comorbidities, specific surgical procedures, and longer hospital stays were associated with lower scores. Fewer than half (102 [47.7%]) achieved functional levels comparable to the 25th percentile of age-matched populations. Multivariate analysis indicated chronic obstructive pulmonary disease and home discharge as predictors of achieving this threshold, while higher PROMIS-PF T-scores were associated with reduced one-year mortality.

Conclusions

Patients undergoing hip fracture surgery are unlikely to achieve high levels of physical function within the three-month postoperative period. Fewer than half of these patients will reach functional levels, and decreased early function is associated with an increased risk of one-year mortality.

## Introduction

Hip fractures and the subsequent surgical treatment significantly influence patient mobility and overall quality of life, as these patients often struggle to achieve their pre-fracture functional ability [1]. These individuals, typically older, confront an added challenge of deconditioning, leading to a loss of ambulation and mobility owing to the weight-bearing nature of the fractured bone [2]. Studies show that approximately one-third will return to their prior level of function, and fewer than a quarter will regain independent mobility [3]. Although mortality rates among this population have been reported to be high and rising by approximately 2% annually as our society ages [4,5], there remains a notable gap in our understanding of the functional outcomes of those who survive these traumatic events. Understanding the determinants of postoperative functional outcomes for these patients is crucial.

The Patient-Reported Outcomes Measurement Information System (PROMIS) was developed in 2004 and funded by the United States National Institutes of Health [6]. It has been reported to be comparable to other patient surveys concerning fracture outcomes in the orthopedic trauma literature [7]. The PROMIS-Physical Function (PF) score holds significant potential for evaluating recovery after hip fractures, as it provides quantifiable measures of patients’ functional status.

To date, a limitation of studies evaluating functional outcomes after hip fractures is the need for external benchmarking against patients of similar ages [8]. Establishing such a benchmark allows for examining the extent to which postoperative functional recovery of hip fracture patients aligns with what might be considered “normal” for the age-matched population. Given the increased baseline fragility of patients presenting with hip fractures, this study specifically focused on comparing this more vulnerable population to the lower 25th percentile of the normal age-matched population. This provides a tangible metric against which the patient-reported outcomes of hip fracture patients can be evaluated, enhancing our understanding of the success and challenges of postoperative rehabilitation. We aimed to explore the factors influencing these postoperative functional outcomes as measured by PROMIS-PF scores reported by hip fracture patients at our institution and to identify predictors of achieving a score equivalent to at least the 25th percentile of the normal age-matched population. Secondarily, we aimed to assess the relationship between three-month postoperative physical function and one-year mortality after hip fracture surgery.

## Materials and methods

Study population

This study was deemed exempt by the institutional review board. A retrospective review of 214 hip and femur fracture patients presenting through the emergency department (ED) from July 2020 to June 2023 was performed. All patients completed a three-month postoperative PROMIS-PF survey. Those who were lost to follow-up (n = 190) or failed to complete a postoperative PROMIS-PF survey (n = 187) at three months postoperatively were excluded. A comparison of the characteristics of excluded and included patients is presented in the Appendix. Patient demographics, comorbidities, procedure performed, time from arrival to surgery, length of stay, and postoperative outcomes were collected.

Outcome measures

The primary outcome of interest was patients’ PROMIS-PF score three months postoperatively. The PROMIS-PF short form 10a was collected at clinic visits. The secondary outcome of interest was mortality at one year postoperatively. The PROMIS-PF questions ask patients to rate the extent to which their health limits the ability to complete various activities including vigorous activities, such as running, lifting heavy objects, participating in strenuous sports; walking more than a mile; climbing one flight of stairs; lifting or carrying groceries; bending, kneeling, or stooping; chores such as vacuuming or yard work; dressing themselves, including tying shoelaces and buttoning clothes; shampooing their hair; washing and drying their body; and sitting on and getting up from the toilet. Individual questions were scored on a 0-5 scale, with higher scores indicating greater levels of functional ability. Aggregate scores were then converted to T-scores, with higher scores again indicating greater levels of functional ability.

Statistical analysis

Descriptive analysis was used to describe patient demographics, comorbidities, procedures performed, time from arrival to surgery, length of stay, postoperative outcomes, and PROMIS-PF overall T-scores and individual question scores. PROMIS-PF scores are normalized across the United States population to a mean of 50 with a standard deviation of 10 [9].

A correlation matrix was created to determine the relationships between the variables collected. Only significant correlations (p < 0.05) were displayed. Multivariate logistic regression was conducted to determine predictors of achieving a PROMIS-PF T-score of least 32.5 at three months postoperatively. The 32.5 cutoff was selected as this was the bottom 25th percentile of PF scores for all orthopedic patients aged 65 to 90 (n = 40,471) in the general population during the study period at our institution. We elected to evaluate this threshold based on prior studies demonstrating the significant impairments to functional abilities after hip fracture surgery. It was, therefore, determined that achieving levels of function above the 25th percentile of the general population was a reasonable goal for hip fracture patients, given their increased baseline fragility.

Univariate analysis and multivariate regression were conducted to determine the relationship between three-month postoperative PROMIS PF T-scores and one-year postoperative mortality. Multivariate regression controlled for age, sex, and the American Society of Anesthesiologists (ASA) scores. All statistical analyses were performed using R Studio (Version 4.2.2 © 2009-2023 RStudio, PBC). Statistical significance was assessed at p-values <0.05.

## Results

Of the 214 patients, the average age was 77.4 years, the average body mass index (BMI) was 24.9 kg/m^2^, and 76.6% of patients were female, while 23.4% were male. Additionally, 72.4% of patients had an ASA score of 3 or greater. The three most prevalent comorbidities were hypertension, diabetes mellitus, and a history of myocardial infarction (MI), which were observed in 56.1%, 22.4%, and 20.1% of patients, respectively. The most common procedure performed was trochanteric femoral nail (TFN) (44.4%), followed by hemiarthroplasty (23.4%), open reduction internal fixation (ORIF) (22.4%), and total hip arthroplasty (THA) (9.8%). The average time to surgery from arrival was 30.2 hours, with 55.1% of patients having surgery within 24 hours of arrival. The average length of stay was 5.2 days, and 29.9% of patients were discharged home. Postoperatively, 7.5% of patients returned to the ED within 90 days for hip soft tissue issues, a fall, pain or weakness, or another medical complication. Additionally, 5.6% of patients were readmitted within 90 days for a re-fracture or another medical complication (Table 1).

**Table 1:** Patients, procedures, and postoperative outcome details. Data are expressed as mean ± SD or n (%). ASA: American Society of Anesthesiologists; BMI: body mass index; ED: emergency department

Demographics	Hip fracture patients (n = 214)
Age, years	77.4 ± 11.7
BMI, kg/m^2^	24.9 ± 5.5
Sex	
Female	164 (76.6)
Male	50 (23.4)
Comorbidities	
ASA score 3+	155 (72.4)
Cerebrovascular disease	9 (4.2)
Congestive heart failure	6 (2.8)
Chronic obstructive pulmonary disease	35 (16.4)
Diabetes mellitus	48 (22.4)
Hypertension	120 (56.1)
Myocardial infarction	43 (20.1)
Pneumonia	6 (2.8)
Peripheral vascular disease	43 (20.1)
Procedures performed	
Total hip arthroplasty	21 (9.8)
Hemiarthroplasty	50 (23.4)
Open reduction internal fixation	48 (22.4)
Trochanteric femoral nail	95 (44.4)
Time from arrival to surgery	
Hours	30.2 ± 16.8
Within 24 hours	118 (55.1)
Length of stay (Days)	5.2 ± 2.4
Discharge home	64 (29.9)
90-day ED return	16 (7.5)
ED return reason	
Fall	2 (1.0)
Hip soft tissue	3 (1.4)
Pain/Weakness	3 (1.4)
Other medical complications	8 (3.7)
90-day readmission	12 (5.6)
Readmission reason	
Re-fracture	3 (1.4)
Other medical complication	9 (4.2)

**Figure 1:**
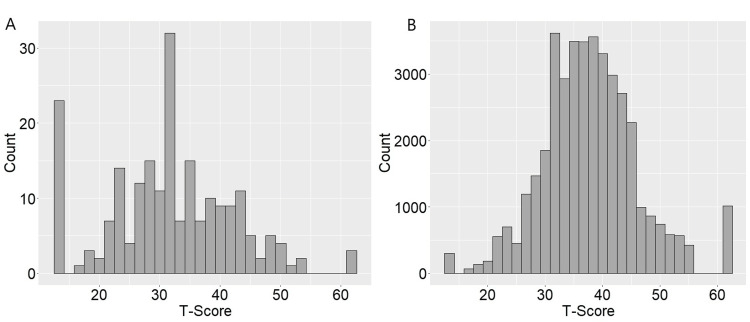
Distribution of PROMIS-PF T-scores. A. Distribution of PROMIS-PF T-scores of hip fracture patients B. Distribution of PROMIS-PF T-scores of all patients aged 65 to 90 years. PROMIS-PF: Patient-Reported Outcome Measurement Information System-Physical Function

The distribution of PROMIS-PF T-scores for the hip fracture patients was skewed to the right with a mean of 31.7 ± 10.3 with a 25th percentile of 25.3 and a 75th percentile of 28.5. The distribution of the normal population aged 65 to 90 years was more normally distributed with a mean of 37.7 ± 8.3 with a 25th percentile of 32.5 and a 75th percentile of 42.6 (Figure 1).

Overall, 47.7% of hip fracture patients achieved levels of physical function at least equivalent to the bottom 25th percentile (PROMIS-PF of ≥32.5) of the general age-matched population.

**Figure 2:**
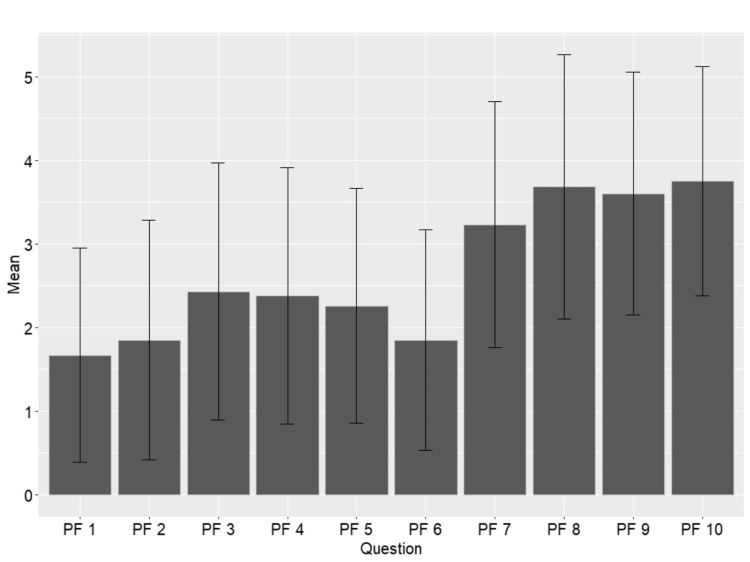
Mean scores of PROMIS-PF questions. Gray bars represent the mean score for each question, with 95% confidence interval indicators; specific questions are further described in the Appendix. PROMIS-PF: Patient-Reported Outcome Measurement Information System-Physical Function

Figure 2 displays the average hip fracture patient score for each specific PROMIS-PF question. The questions that had the highest average scores were 7-10. These questions surround self-care and independent tasks such as dressing yourself and showering. The questions with the lowest average scores were 1-6, which surrounded vigorous activities such as running, walking, lifting, and house or yard work.

**Figure 3:**
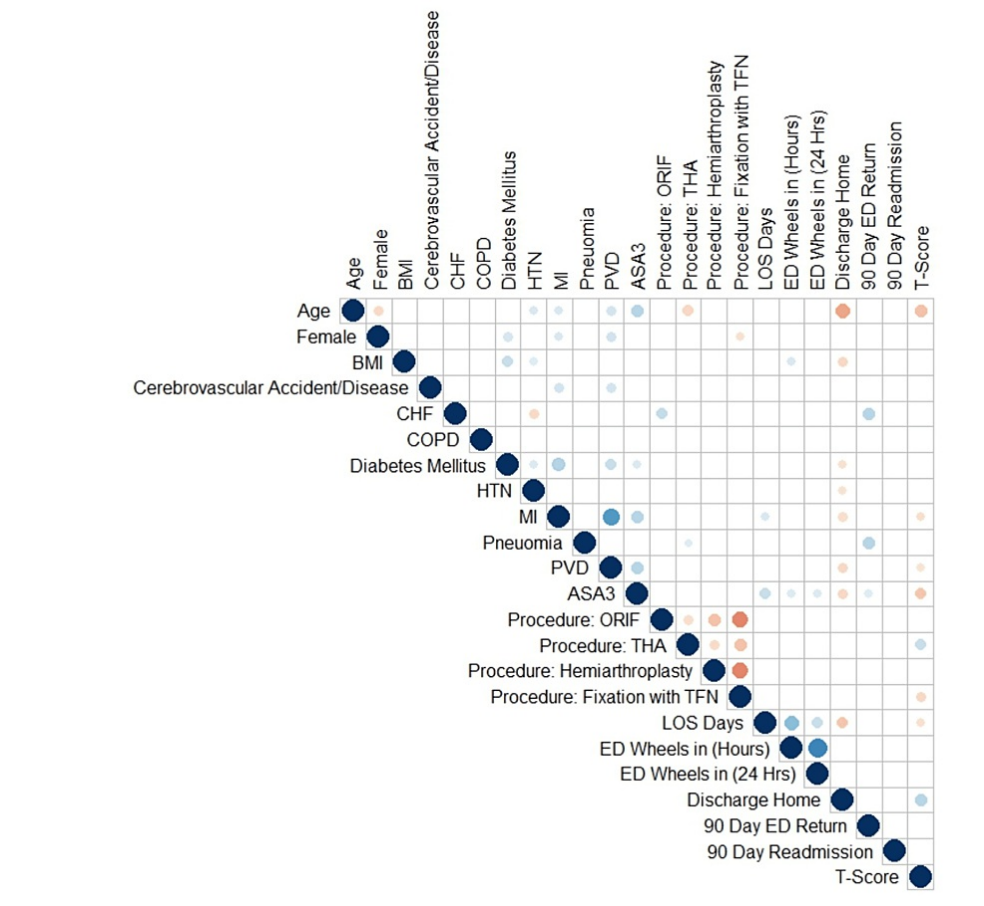
Correlation matrix of all variables. Blue dots indicate significant positive correlations and orange dots indicate significant negative correlations with the larger and deeper colored dots indicating a greater degree of correlation. ASA3: American Society of Anesthesiologists score 3 or higher; BMI: body mass index; CHF: congestive heart failure; COPD: chronic obstructive pulmonary disease; ED: emergency department; HTN: hypertension; LOS: length of stay; MI: myocardial infarction; PVD: peripheral vascular disease; ORIF: open reduction internal fixation; THA: total hip arthroplasty; TFN: trochanteric femoral nail

In multivariable logistic regression, THA and home discharges were associated with higher physical function scores postoperatively. Conversely, increased age, ASA scores ≥3, a history of MI or peripheral vascular disease (PVD), TFN fixation, and longer length of stay were associated with lower PROMIS-PF scores postoperatively (Figure 3).

Multivariate logistic regression showed that only chronic obstructive pulmonary disease (COPD) and discharge to home were predictive of achieving at least the bottom 25th percentile PROMIS-PF T-score of the normal population aged 65 to 90 years. A patient with COPD was 2.70 (odds ratio [OR] = 0.37, 95% confidence interval [CI] 0.15 to 0.86; p = 0.024) times less likely to reach at least the bottom 25th percentile, while a patient who was discharged home was 3.21 (OR = 3.21, 95% CI = 1.46 to 7.32; p = 0.004) times more likely to reach at least the bottom 25th percentile T-score within three months postoperatively. However, this model only explained 20.9% of the variability in the achievement of at least the bottom 25th percentile score (R^2^ = 0.209) indicating that additional unmeasured factors account for approximately 80% of postoperative physical function (Table 2).

**Table 2:** Predictors of achieving 25th percentile PROMIS-PF T-score of the normal population. Multivariate logistic regression; P-values <0.05 in bold ASA: American Society of Anesthesiologists; BMI: body mass index; ED: emergency department: PROMIS-PF: Patient-Reported Outcome Measurement Information System-Physical Function

Demographics	Odds ratio	95% confidence interval	P-value
Age, years	0.99	0.96 to 1.03	0.720
BMI, kg/m^2^	1.00	0.94 to 1.06	0.963
Female	1.82	0.82 to 4.14	0.146
Comorbidities			
ASA score 3+	0.49	0.22 to 1.06	0.072
Cerebrovascular disease	1.32	0.26 to 6.39	0.730
Congestive heart failure	0.36	0.04 to 2.50	0.317
Chronic obstructive pulmonary disease	0.37	0.15 to 0.86	0.024
Diabetes mellitus	0.76	0.32 to 1.76	0.522
Hypertension	0.62	0.32 to 1.19	0.151
Myocardial infarction	1.16	0.44 to 3.02	0.763
Pneumonia	0.45	0.05 to 3.01	0.427
Peripheral vascular disease	0.59	0.22 to 1.52	0.280
Procedures performed			
Total hip arthroplasty	2.39	0.95 to 6.54	0.073
Hemiarthroplasty	0.85	0.23 to 2.95	0.793
Open reduction internal fixation	0.85	0.23 to 3.03	0.807
Trochanteric femoral nail	0.39	0.12 to 1.23	0.114
Time from arrival to surgery			
Hours	1.00	0.97 to 1.03	0.876
Within 24 hours	0.96	0.40 to 2.27	0.917
Length of stay (days)	1.01	0.86 to 1.18	0.933
Discharge home	3.21	1.46 to 7.32	0.004
90-day ED return	1.46	0.43 to 4.99	0.541
90-day readmission	0.29	0.06 to 1.17	0.099
R^2^ = 0.209			

Univariate analysis showed that patients who died within one year postoperatively had a lower three-month postoperative PROMIS PF T-score than those who did not die within one year of their surgery (20.0 ± 7.4 vs. 32.9 ± 9.8; p < 0.001). Multivariate analysis showed that each one-point increase in PROMIS PF T-scores at three months postoperatively was associated with 13% reduced odds of one-year mortality (OR = 0.87, 95% CI = 0.80 to 0.93; p < 0.001). Additionally, patients whose PROMIS PF T-score was below average (T-score less than 31.7) at three months postoperatively were 5.66 times more likely to die within one year of surgery (OR = 5.66, 95% CI = 1.67 to 9.39; p = 0.011) (Table 3).

**Table 3:** Three-month PROMIS-PF T-scores and one-year mortality. P-values <0.05 in bold; ^a^: controlling for age, sex, and ASA score 3+. ASA: American Society of Anesthesiologists; PROMIS-PF: Patient-Reported Outcome Measurement Information System-Physical Function

Multivariate ^a^	Odds ratio	95% confidence interval	
PROMIS PF T-score	0.87	0.80 to 0.93	<0.001
Below mean T-score (<31.7)	5.66	1.67 to 9.39	0.011

## Discussion

Each year, more than 350,000 people in the United States suffer from hip fractures, making these injuries a significant burden to both individual patients and the healthcare system [8]. Identifying factors influencing postoperative functional outcomes offers predictive insights into achieving scores analogous to the lower quartile of age-matched populations and are associated with one-year mortality following hip fracture surgery. These findings provide essential benchmarks, enabling the establishment of realistic expectations for patients and families grappling with hip fracture injuries.

Our findings resonate with the conclusions drawn in existing studies regarding hip fracture patients’ challenges in achieving normal physical function. The adverse effects of hip fractures on patient quality of life have been described by Burns et al., who found that 26% of hip fracture patients reported poor mobility, high levels of support care requirement, and death, and 16% were found to be depressed [10]. Hip fracture patients also exhibit increased adverse events and a heightened likelihood of requiring ambulance transport following their discharge from the hospital [11]. Even patients under 65 years old, though they have higher survival rates, still report poor functional outcomes [12].

In the current study, factors such as advanced age, higher ASA classification, MI, PVD, and prolonged length of stay were negatively associated with PROMIS-PF T-scores, suggesting that these factors may impede recovery and functional outcomes. These findings are in alignment with a 2019 systematic review that also found that medical factors such as a history of MI, PVD, heart failure, depression, visual impairment, and frailty correlate with poorer outcomes in patients with hip fractures [13]. Similar trends have been observed within the elective THA population, as advanced age and elevated ASA scores have been demonstrated to be predictors of impaired clinical and patient-reported outcomes [13-15]. Collectively, these findings reinforce the notion that patients with significant medical comorbidities are unlikely to achieve high levels of functional recovery and should be counseled regarding this point.

Hip fractures have been known to result in significant mortality as well, with studies showing as much as a 1.8-year life reduction after a hip fracture [16]. Patients in this study who did not survive one year postoperatively exhibited lower PROMIS PF T-scores three months after surgery, underscoring the potential significance of early postoperative physical function in predicting long-term outcomes. The multivariate analysis further corroborated this observation, as patients with lower physical function scores at three months postoperatively were at an increased risk of one-year mortality after adjusting for other confounding factors. While a multitude of factors influence postoperative survival, these results highlight the potential prognostic utility of early physical function measurement that may further assist with patient and family expectation setting.

According to current guidelines, surgical treatment of hip fractures should be performed within the first 24 hours following the injury [17]. This early intervention has been linked to reduced length of stay, decreased pain duration, and lower occurrences of nonunion, postoperative complications, and mortality [17-19]. A recent study by Liu et al. found that hip fracture surgery performed within two days of injury resulted in improved surgical and medical outcomes [19]. Darbandi et al. found that delayed surgery increased mortality by 1.475-fold and increased length of stay, readmission rates, and medical complications [20]. However, there has been limited research on the correlation between surgical timing and functional outcomes. While some studies propose improved functional outcomes with early surgical intervention [21], conflicting results have been reported in others [22]. While we did observe a statistically significant negative association between length of stay and postoperative PROMIS-PF scores, we did not find the overall length of stay, time from arrival to surgery, or surgery within 24 hours of arrival to be independent predictors of physical function levels at three months postoperatively. Based on these findings, we suggest that while pathways aimed at providing efficient surgical treatment to hip fracture patients remain important for minimizing the risk of postoperative complications, they are unlikely to translate to differences in functional recovery over the longer term.

The questions within the PROMIS-PF survey provided valuable insights into the specific areas of functional limitation. Notably, questions related to vigorous activities, such as running, walking, and lifting, garnered lower average scores, reflecting challenges in these domains post-hip fracture repair. Conversely, questions centered around self-care and independent tasks, such as dressing and showering, yielded higher average scores, indicating a greater ability to perform these activities. While other studies have found that only 40-60% of hip fracture patients achieve their pre-injury mobility and daily living capabilities [1], the results of the current study provide further insights into which types of abilities patients may expect to recover postoperatively. Based on our results, we suggest patients and families be counseled that efforts to restore the ability to perform self-care activities should be the focus during recovery, as it is unlikely that patients will achieve high levels of mobility.

Our predictive analysis demonstrated that two independent factors, COPD and discharge to home, significantly influenced the likelihood of achieving a PROMIS-PF T-score comparable to the 25th percentile of the normal population. Interestingly, patients with COPD exhibited a decreased probability of achieving this benchmark score, highlighting the impact of respiratory health on postoperative recovery, as demonstrated by previous studies [23,24]. Yakubek et al. found that COPD patients had longer lengths of stay and were more likely to suffer postoperative complications following THA [25]. In contrast, patients who were discharged home after surgery were significantly more likely to attain a T-score closer to the normal population. While it is likely that the ability to be discharged home reflects overall patient health and functional status before suffering a hip fracture, this result underscores the importance of offering resources and assistance to facilitate the return of as many patients as possible to their homes following acute treatment. Kimmel et al. observed similar results in their prospective study, noting that discharge to inpatient rehabilitation after treatment for isolated lower limb fractures was associated with poorer outcomes than discharge home [26]. However, discharge plans are dependent on multiple factors, and in some instances, home discharge may not be the best option. Other studies have found that discharge to home was associated with higher rates of readmission and rehospitalization compared with discharge to a skilled nursing facility, sometimes with no difference in functional outcomes [27,28]. Ultimately, while our results demonstrate that home discharge is associated with improved postoperative physical function and should be pursued, when possible, decisions regarding optimal discharge destination must continue to be made on a patient-specific basis.

This study has certain limitations, including its retrospective nature and the potential presence of unmeasured confounding variables. Additionally, the study was conducted within a single institution and focused on a specific geographic region, potentially limiting the generalizability of the findings to a broader population of individuals with hip fractures. Another limitation lies in the selection of the 25th percentile of the normal population as the threshold for targeted improvement. While the selection of this outcome was inherently subjective, it is an appropriate postoperative function target that is realistically achievable for hip fracture patients, given that approximately 48% achieved this level of function postoperatively. Moreover, the term “normal population” in this context refers to a patient cohort being treated for orthopedic conditions, which may result in lower functional scores compared to entirely healthy individuals of the same age. An additional potential source of bias stems from the inclusion criteria, which only encompassed patients who were able to return for follow-up surveys. This may lead to an overestimation of functional outcomes, as those unable to return for follow-up may have lower functional capabilities. Although we were unable to directly assess the functional outcomes of patients who were lost to follow-up or did not complete the PROMIS surveys, a comparison of baseline demographics, comorbidities, and types of procedures performed demonstrated that these patients were largely similar to those included in the study. As shown in the Appendix, excluded patients were approximately four years older than included patients on average, while included patients were more likely to have hypertension; no significant differences in BMI, sex, ASA classification, other comorbidities, or types of procedures performed existed between these populations. Therefore, the patients included in the study accurately represent hip fracture patients at our institution. Finally, the short-term follow-up period employed in this study may not capture the patients’ true long-term level of function.

## Conclusions

Patients undergoing hip fracture surgery are unlikely to achieve high levels of physical function within the three-month postoperative period. Fewer than half of these patients will reach functional levels in alignment with the bottom 25% of the general population of similar age, and decreased early function is associated with an increased risk of one-year mortality. Although relationships between patient characteristics, hospital course, and functional outcomes exist, these appear to be relatively weak and of limited ability to be modified. While further studies of interventions that may improve postoperative function are warranted, these findings provide important benchmarks that may be used to establish realistic expectations for both patients and families.

## Appendices

**Table 4:** Univariate comparison of demographics, comorbidities, and procedures performed: excluded vs. included patients. P-values <0.05 in bold; data are expressed as mean ± SD or n (%). ASA: American Society of Anesthesiologists; BMI: body mass index

Patient characteristic	Excluded patients (n = 377)	Included patients (n = 214)	P-value
Age, years	81.1 ± 10.8	77.4 ± 11.7	<0.001
BMI, kg/m^2^	24.4 ± 5.1	24.9 ± 5.5	0.259
Female	262 (69.5)	164 (76.6)	0.077
ASA score 3+	293 (77.7)	155 (72.4)	0.179
Coronary artery disease	14 (3.7)	9 (4.2)	0.939
Congestive heart failure	8 (2.1)	6 (2.8)	0.809
Chronic obstructive pulmonary disease	76 (20.2)	35 (16.4)	0.304
Diabetes	71 (18.8)	48 (22.4)	0.347
Hypertension	167 (44.3)	120 (56.1)	0.008
Myocardial infarction	77 (20.4)	43 (20.1)	1
Pneumonia	8 (2.1)	6 (2.8)	0.809
Peripheral vascular disease	73 (19.4)	43 (20.1)	0.915
Procedure performed			0.215
Trochanteric femoral nail	191 (50.7)	98 (45.8)	
Hemiarthroplasty	99 (26.3)	50 (23.4)	
Open reduction internal fixation	62 (16.4)	45 (21.0)	
Total hip arthroplasty	25 (6.6)	21 (9.8)	

PROMIS-PF questions

1.      Does your health now limit you from doing vigorous activities, such as running, lifting heavy objects, and participating in strenuous sports?

2.      Does your health now limit you from climbing one flight of stairs?

3.      Does your health now limit you to walking more than a mile?

4.      Does your health now limit you in lifting or carrying groceries?

5.      Does your health now limit you in bending, kneeling, or stooping?

6.      Are you able to do chores such as vacuuming or yard work?

7.      Are you able to dress yourself, including tying shoelaces and doing buttons?

8.      Are you able to shampoo your hair?

9.      Are you able to wash and dry your body?

10.    Are you able to get on and off the toilet?
